# Tumor Imaging and Targeting Potential of an Hsp70-Derived 14-Mer Peptide

**DOI:** 10.1371/journal.pone.0105344

**Published:** 2014-08-28

**Authors:** Mathias Gehrmann, Stefan Stangl, Gemma A. Foulds, Rupert Oellinger, Stephanie Breuninger, Roland Rad, Alan G. Pockley, Gabriele Multhoff

**Affiliations:** 1 Department of Radiation Oncology, Klinikum rechts der Isar, Technische Universität München, Munich, Germany; 2 John van Geest Cancer Research Centre, Nottingham Trent University, Nottingham, United Kingdom; 3 Medical Department II, Translational Gastroenterological Oncology, Klinikum rechts der Isar, Technische Universität München, Munich, Germany; 4 Clinical Cooperation Group (CCG) ‘‘Innate Immunity in Tumor Biology’’, Helmholtz Zentrum München, Deutsches Forschungszentrum für Gesundheit und Umwelt, Munich, Germany; Boston University Goldman School of Dental Medicine, United States of America

## Abstract

**Background:**

We have previously used a unique mouse monoclonal antibody cmHsp70.1 to demonstrate the selective presence of a membrane-bound form of Hsp70 (memHsp70) on a variety of leukemia cells and on single cell suspensions derived from solid tumors of different entities, but not on non-transformed cells or cells from corresponding ’healthy‘ tissue. This antibody can be used to image tumors *in vivo* and target them for antibody-dependent cellular cytotoxicity. Tumor-specific expression of memHsp70 therefore has the potential to be exploited for theranostic purposes. Given the advantages of peptides as imaging and targeting agents, this study assessed whether a 14-mer tumor penetrating peptide (TPP; TKDNNLLGRFELSG), the sequence of which is derived from the oligomerization domain of Hsp70 which is expressed on the cell surface of tumor cells, can also be used for targeting membrane Hsp70 positive (memHsp70+) tumor cells, *in vitro*.

**Methodology/Principal Findings:**

The specificity of carboxy-fluorescein (CF-) labeled TPP (TPP) to Hsp70 was proven in an Hsp70 knockout mammary tumor cell system. TPP specifically binds to different memHsp70+ mouse and human tumor cell lines and is rapidly taken up via endosomes. Two to four-fold higher levels of CF-labeled TPP were detected in MCF7 (82% memHsp70+) and MDA-MB-231 (75% memHsp70+) cells compared to T47D cells (29% memHsp70+) that exhibit a lower Hsp70 membrane positivity. After 90 min incubation, TPP co-localized with mitochondrial membranes in memHsp70+ tumors. Although there was no evidence that any given vesicle population was specifically localized, fluorophore-labeled cmHsp70.1 antibody and TPP preferentially accumulated in the proximity of the adherent surface of cultured cells. These findings suggest a potential association between membrane Hsp70 expression and cytoskeletal elements that are involved in adherence, the establishment of intercellular synapses and/or membrane reorganization.

**Conclusions/Significance:**

This study demonstrates the specific binding and rapid internalization of TPP by tumor cells with a memHsp70+ phenotype. TPP might therefore have potential for targeting and imaging the large proportion of tumors (∼50%) that express memHsp70.

## Introduction

Significant progress in the development of new therapies that can increase overall survival rates for a range of cancer types has been made. However, the heterogeneity within individual tumors and between tumors of the same type in different patients, as well as the different stages and sub-types of tumors, combine to confer a level of resistance to existing treatments in patients, and problems with the application of universal treatment strategies. Although chemotherapy remains one of the primary approaches for treating cancer, conventional therapy is not specifically targeted to tumor cells and is therefore associated with a high level of side-effects, some of which can be severe. The development of drug resistance and other issues associated with biodistribution and drug clearance also pose significant problems and challenges.

The difficulties in effectively targeting tumors that result from genetic and phenotypic heterogeneity have prompted the need for more specifically targeted approaches - ‘patient stratification’ and ‘patient-focused medicine’. However, the ability to better treat a patient on the basis of the characteristics of their own tumor requires the identification of key target molecules or features. Although the ability to better identify tumor-associated antigens has led to the development of engineered human monoclonal antibodies for targeting a wide range of common malignancies [1] and the use of radiolabeled antibodies as therapeutics for different kinds of hematological malignancies and solid tumors [2]–[5], the production and purification of therapeutic antibodies is time-consuming and cost-intensive. Furthermore, antibodies derived from mice are not suitable for therapeutic approaches due to their immunogenicity in humans, and the affinity, avidity, or even the specificity of chimeric humanized antibodies often differ from those of the original murine antibody from which they are derived.

The primary advantage of antibodies is their specificity and ability to target single molecules bearing the epitope. However, this can also be a disadvantage, as antibodies can only target those cancers which express the antigenic determinant. It is also the case that the identification of such targets does not always result in an effective treatment. As an example, only a proportion (∼50%) of those patients that bear Her2-positive breast tumors and are therefore theoretically sensitive to trastuzumab (Herceptin), respond to the drug, and many tumors develop resistance to treatment [6].

Attention is now being turned towards the use of small molecules such as peptides for diagnostic imaging and targeted radionuclide therapy, due to the fact that they are easy to produce with a defined and controlled purity in large quantities. Such peptides exhibit a better biodistribution compared to antibodies are generally more stable and their enhanced ability to penetrate tissues makes them better targeting agents. Peptides can be specifically taken up via specialized receptors [7], [8], and therapeutic peptides have been used in the treatment of breast [9] and other types of cancer [10]. Peptides, such as somatostatin, bombesin, cholecystokinin/gastrin, neurotensin and vasoactive intestinal peptide are currently under investigation for their possible clinical applications in nuclear oncology [11]. Despite the potential of these molecules, it remains essential that more universally expressed molecules which can be used as recognition structures for imaging and targeting agents are identified. One recognition structure which has significant potential as a target recognition structure in cancer is a membrane form of the 70 kDa heat shock (stress) protein family of molecules (heat shock protein 70, Hsp70, HSPA1A) [12]–[16].

Hsp70 is a cytoprotective molecule which is constitutively overexpressed in the cytoplasm of tumor cells [17]. Tumor cells overexpressing Hsp70 are resistant to radiation and cytostatic drugs [18]. In addition to being an intracellular molecule, we have previously reported that Hsp70 can also be selectively expressed on the plasma membrane of tumor cells using a unique mouse monoclonal antibody (clone cmHsp70.1, multimmune GmbH) which specifically recognizes the membrane form of Hsp70 [12]–[16], [19], [20]. The membrane expression of Hsp70 is independent of endoplasmic reticulum (ER) or Golgi apparatus dependent pathways [21], but does appear to be influenced by the lipid composition of the plasma membrane [22].

This membrane form of Hsp70 is widely expressed on cultured cancer cells, including leukemic cells [23], [24], lung [12], colorectal [16], [25], and breast cancer cells [19]. Furthermore, an ongoing screening program which currently includes the analysis of primary tumors from over 1,000 patients has demonstrated that this membrane form of Hsp70 is also present on approximately 50% of all tumor entities tested, including leukemia [26], [27], melanoma [28], gastrointestinal [25] and breast tumors [29]. In contrast, cells from adjacent and other healthy tissues do not express the membrane form of Hsp70 [12]–[16]. Membrane-Hsp70 therefore appears to offer an ideal target for diagnostic, prognostic, and therapeutic approaches.

Previous experiments have shown that the cmHsp70.1 antibody can be taken up by the memHsp70+ murine colorectal cancer cell line CT26 and that its intracellular transportation occurs via endosomal pathways [30]. Furthermore, the cmHsp70.1 antibody can be used to image memHsp70+ tumors *in vivo* [20] and can induce antibody-dependent cellular cytotoxicity (ADCC) of memHsp70+ mouse tumor cells [19]. MemHsp70 also acts as a target recognition structure for recombinant human granzyme B, which is internalized and induces apoptosis without a requirement for perforin [29]–[31]. More recently, we observed that an Hsp70-derived 14-mer peptide (TPP) specifically binds to memHsp70+ tumor cells. Preliminary experiments revealed that the binding to Hsp70 is followed by a rapid and quantitative internalization of TPP, whereas scrambled peptides were not internalized. Since TPP is part of the oligomerization domain of Hsp70, we hypothesize that the binding of TPP to Hsp70 involves mechanisms that are related to the oligomerization domain of Hsp70. Furthermore, it appeared that the uptake and intracellular transport of TPP follows similar pathways as those for the cmHsp70.1 antibody.

Given the biodistribution advantages of peptides, this study examined the binding and internalization of the TPP to memHsp70+ cancer cell lines, *in vitro*. The specific binding of the TPP to tumor cells expressing the membrane form of Hsp70 and its uptake into these cells which is demonstrated indicates that this peptide is a promising imaging and therapeutic vehicle for targeting memHsp70+ tumor cells *in vivo*.

## Materials and Methods

### Cell lines

Human MCF7 (ATCC HTB-22), MDA-MB-231 (ATCC HTB-26), T47D (ATCC HTB-133) breast, CX-2 (TZB 61005), HCT116 (ATCC CCL-247) colon, Mel Ho (DSMZ ACC-62), Parl melanoma (kindly provided by Prof. J. Johnson, Institute of Immunology, Ludwig-Maximilians-Universität München, Munich), A549 (DSMZ MACC-107), H1339 (DSMZ ACC-506), EPLC-272H (DSMZ ACC-383) lung, FaDu (ATCC HTB-43) squamous carcinoma of the head & neck, and mouse 4T1 breast (4T1 wild type, 4T1 Hsp70^−/−^ knock-out) cancer cell lines were grown in appropriate cell culture medium under standard conditions (37°C, 95% v/v humidity, 5% v/v CO_2_) and passaged twice a week. Cells were used in exponential phase and their viability (>95%) confirmed prior to use using trypan blue dye exclusion. Cells were routinely tested to ensure the absence of mycoplasma.

### Proteins and peptides

Recombinant His-tagged Hsp70 protein, which is equivalent to HSPA1A (P08107), was isolated from transfected Sf9 insect cells (Orbigen, San Diego, CA, U.S.A.), as described elsewhere [32]. Other recombinant HSP proteins including Hsp60 (ADI-NSP-540), Grp78 (ADI-SPP-765), Hsp27 (ADI-SPP-715) were obtained from Assay Designs (Ann Arbor, MI, U.S.A., now Enzo Life Sciences). Carboxyfluorescein (CF-) labeled 14-mer peptides TKDNNLLGRFELSG (TPP) and the scrambled peptide (scrambled) with identical amino acid composition NGLTLKNDFSRLEG were obtained from EMC microcollections (Tuebingen, Germany) at a purity >97%. Lyophilized peptides were reconstituted to a stock concentration of 1 mg/ml using distilled water and stored at 4°C for a period of no more than two weeks.

### Antibodies

The expression of the membrane form of Hsp70 was determined using the memHsp70 specific murine monoclonal antibody cmHsp70.1-FITC (IgG1; multimmune GmbH, Germany). Monoclonal antibodies recognizing Rab4, Rab5, Rab7, Rab9 and Rab11 were obtained from Santa Cruz Biotechnology (Santa Cruz, CA, U.S.A.) and antibodies recognizing LAMP1 and LAMP2 were obtained from Sigma-Aldrich (St. Louis, MO, U.S.A.). Appropriate Cy3-conjugated anti-goat and anti-rabbit immunoglobulin secondary antibodies were obtained from Jackson ImmunoResearch (Newmarket, UK).

### Peptide and antibody binding - flow cytometry

The expression of memHsp70 on tumor cell lines was determined by flow cytometry using either the FITC-conjugated cmHsp70.1 antibody or the CF-labeled 14-mer peptide TPP, both of which bind to the exposed sequence of memHsp70. Briefly, after incubation of viable cells (2×10^5^ cells) with the antibody or peptide for 30 min at 4°C and following two washing steps, viable (propidium iodide negative) cells were analyzed using a FACSCalibur flow cytometer (BD Biosciences, Franklin Lakes, NJ, U.S.A.). An isotype-matched (IgG1) control antibody (BD Biosciences) was used to evaluate non-specific binding to cells.

### Hsp70 knockout – CRISPR/Cas9 knockout of the genes Hspa1a and Hspa1b

Knockout of the neighbouring and closely homologous genes Hspa1a (ENSMUSG00000091971) and Hspa1b (ENSMUSG00000090877) in 4T1 tumor cells was achieved by double nicking of closely adjacent sites with CRISPR/Cas9. This DNA damage leads to DNA repair of the NHEJ type and generates small insertions or deletions (indel formation) that often results in frameshifts within the coding sequence (cds), and therefore knockout of gene expression [33].

To target both genes we selected two guide sequences using the CRISPR design tool [34] (guide-1 GATGCCGATCGCCGTGTTCT; guide-2 GCACGGCGATCGGCATCGACC) that target the 5’ region of the cds of both genes and cloned those into the vector pX462. 4T1 cells were transfected with a mixture of both vectors using Lipofectamine 2000 (Life Technologies, 250 ng each in a 24 well plate) and the transfected cells were put under puromycin selection (2.5 µg/ml) for two days. Single cells clones were generated by limiting dilution assays and tested for knockout of Hspa1a and Hspa1b expression with Western Blot analysis.

### Western Blot analysis

Cells were lysed in TBST buffer (1% Triton X-100 in TBS, 1 mM PMSF, protease inhibitor cocktail) and protein content determined using the BCA Protein Assay kit (Pierce). For Western Blot analysis, proteins were detected with monoclonal antibodies directed against Hsp70 (cmHsp70.1, multimmune GmbH) and β-actin (Sigma). Bound antibodies were visualized using horseradish peroxidase-conjugated secondary antibodies (Dako) and a chemiluminescence developing kit (ECL, Amersham Biosciences).

### Peptide ELISA

The *in vitro* binding of peptides to heat shock proteins was determined using a peptide ELISA. Briefly, Hsp70, Hsp60, Grp78, and Hsp27 proteins were coated onto 96-well MaxiSorp plates (Thermo Fisher Scientific, Roskilde, Denmark) at a concentration of 1 µg/well/100 µl in carbonate buffer (pH 9.6) at 4°C overnight. After washing, wells were blocked with phosphate buffered saline (PBS) containing 2% w/v bovine serum albumin (BSA) at room temperature for 2 h. Blocking buffer was discarded and wells were incubated with CF-labeled peptides (100, 50, 25 ng/ml) in a total volume of 100 µl at 27°C for 30 min. After another washing step the fluorescence resulting from specifically bound peptides was measured using a Victor X4 Multilabel Plate Reader (PerkinElmer, Waltham, MA, U.S.A.) equipped with appropriate filters.

### Peptide uptake – flow cytometry

Cells were grown in T75 flasks for 48 h, at which time they were harvested using trypsin for 1 min at 37°C and counted using trypan blue dye exclusion. Viable cells (1×10^6^ cells) were transferred into 1.5 ml microfuge tubes and washed with PBS (300 g, 5 min). CF-labeled TPP (20 µl, 75 µg/ml in PBS) was added to the cells and then the cell/peptide mixture was divided into two microfuge tubes (10 µl in each). One tube was kept on ice and the other put into the 37°C incubator. At the indicated time points (0, 5, 15, 30, 60 min), an aliquot of the cell suspension (2 µl) was transferred into 12×75 mm tubes containing 3 ml of chilled PBS. After washing twice (300 g, 5 min), cells were suspended in 250 µl chilled PBS at 4°C and analyzed on a BD FACSCalibur flow cytometer. Propidium iodide (PI) was added immediately prior to flow cytometric analysis in order to exclude non-viable cells from the analysis. Additionally, after incubation with TPP or scrambled control peptide (7.5, 75, or 750 µg/ml) for 24, 48, or 72 h cell viability was tested with the FITC Active Caspase-3 Apoptosis kit (BD Biosciences) or FlowCellect Cytochrome c kit (EMD Millipore Corporation, Hayward, CA, U.S.A.).

Cells for analysis were identified on the basis of forward and side light scatter characteristics (FSC, SSC respectively) and confirmed as being single cells using the FSC-A(rea) and SSC-H(eight) parameters. Peptide uptake into viable cells was determined on the basis of the fluorescence intensity of the cell population.

### Peptide uptake – confocal microscopy

Cells were grown in MatTek (Ashland, MA, U.S.A.) dishes for 48 h. Diluted peptide (100 µl, 75 µg/ml) was added to cells and the dishes were incubated at 37°C for 30 min. Cells were washed in 2 ml PBS at 4°C then fixed with 0.4% w/v paraformaldehyde (Sigma-Aldrich). Coverslips were detached by incubating dishes in 750 µl removal fluid (MatTek) for 20 min. The coverslips were then mounted onto clean microscopy slides using Vectashield Medium containing DAPI (Vector Laboratories, Burlingame, CA, U.S.A.). Coverslips were sealed using clear nail varnish and the slides were kept cool and protected from light until imaging could commence. Cells were imaged on a Zeiss Inverted 510 LSM microscope (Carl Zeiss AG, Oberkochen, Germany). A single frame overview was produced with the pinholes open, from which individual cells were selected for z-stack imaging. The single frame image was produced using a 20×/0.8 dry objective at 2048×2048 resolution with 16× mean averaging. Z-stack images were obtained using a 63×/1.4 oil immersion objective at 2048×2048 resolution and 8× mean averaging.

### Transfection of breast cancer cell lines

Co-localization of CF-labeled peptides with intracellular vesicles was determined using breast cancer cells which had been transfected to express red fluorescent protein (RFP) tagged marker proteins for early endosomes (Rab5), late endosomes (Rab7), or lysosomes (LAMP1) using ‘CellLight Reagents *BacMam 2.0*’ according the manufacturers’ instructions (Molecular Probes, Life Technologies, Carlsbad, CA, U.S.A.). Briefly, cells were grown for 24 h to 50% confluency in MatTek chamber slides (Thermo Scientific, Rochester, NY, U.S.A.). The medium was removed and replaced with fresh medium containing 2 µg/ml transfection reagent with baculovirus containing sequences for the expression of RFP tagged marker proteins for Rab5, Rab7, or LAMP1. RFP could be detected in 70–90% of the cells 24 to 48 h after transfection and the staining was stable for at least 60 h in the cell lines used.

### Peptide uptake – fluorescence microscopy

Cells transfected with endosomal or lysosomal markers were grown in chamber slides (Thermo Scientific, Rochester, NY, U.S.A.) for 24 h. Diluted CF-labeled peptide (75 µg/ml, 100 µl) was added to cells and the chamber slides were incubated at 37°C for the indicated time periods. Cells were washed three times with 0.5 ml PBS and imaged on a Zeiss Axio Observer Z1 microscope (Carl Zeiss AG, Oberkochen, Germany). Z- and t-stack images were measured with a 63× long-distance objectives for different time periods and different settings. Images were taken in brightfield and FITC-, and RFP-channels. Multi-color images were produced by merging.

### Imaging and software

Images were taken and processed using AxioVision Software, LSM Image Browser (Carl Zeiss AG, Oberkochen, Germany), or ImageJ (NIH, Bethesda, MD, U.S.A.) [35]. Image processing consisted of brightness and contrast adjustments, as well as running a noise reducing despeckling algorithm. Both Pearson’s coefficient and Manders’ M1 coefficient co-localization analyses [36] were conducted using the ‘*Just Another Co-localization Program*’ (JACoP) plugin on ImageJ [37]. Further image processing - splitting each image into separate channels and setting fluorescence thresholds - was required prior to undertaking the co-localization analyses.

### Statistics

The Student’s *t*-test or factorial ANOVA were used, as appropriate, with the significance level set to *p*<0.05. Evaluation of the co-localization of CF-labeled TPP with mitochondria was calculated using Manders’ M1 coefficient and Pearson’s coefficient.

### Miscellaneous

Chemicals or other material were obtained from Sigma Aldrich or Carl Roth GmbH (Karlsruhe, Germany).

## Results

### Tumor cell lines expressing membrane-Hsp70 (memHsp70) bind TPP

**Figure 1:**
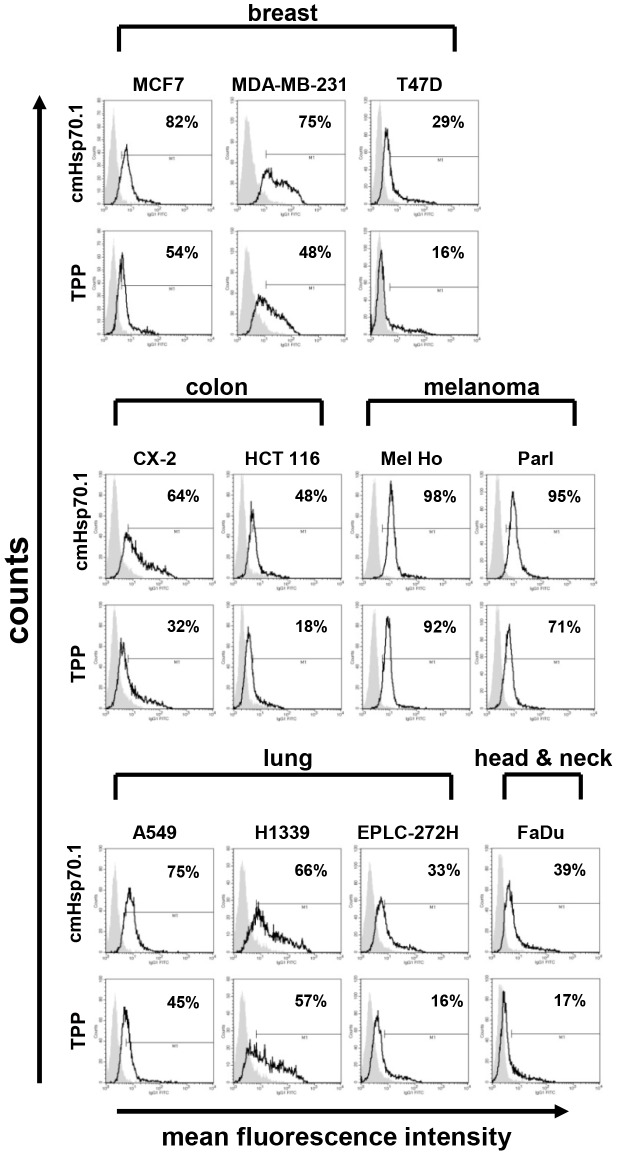
Tumor cell lines expressing memHsp70 are able to bind TPP at 4°C. The memHsp70 status of a panel of human breast, colon, melanoma, lung, head & neck tumor cell lines was assessed using the cmHsp70.1 antibody (top row histograms). Incubation of tumor cells with carboxyfluorescein (CF)-labeled TPP at 4°C (bottom row histograms) results in a similar binding profile to that of cmHsp70.1 antibody in all tumor cell lines; grey, isotype controls, open histograms, Hsp70 specific reagents. The numbers in the histograms show the proportion of Hsp70 membrane-positively stained cells.

MemHsp70 expression by different human tumor cell lines and the relationship between memHsp70 expression and the ability to bind a 14-mer peptide TPP matching an epitope within the oligomerization domain of the Hsp70 molecule (aa 450–463 TKDNNLLGRFELSG, abbreviation TPP) were determined by flow cytometry at 4°C (**Fig. 1**). MemHsp70 is demonstrated on the basis of the mean fluorescence intensity of cmHsp70.1 antibody staining (open histograms in the top row of each display), with the proportion of cells exhibiting positive staining given in the upper right corner of each histogram (**Fig. 1**). The expression profiles varied between the different cell lines, even cells that have been derived from the same tumor entity. The flow cytometry experiments were all controlled using appropriate isotype-matched reagents (grayed histogram).

The binding profile of carboxyfluorescein (CF)-labeled TPP at 4°C was similar to that of the cmHsp70.1 antibody for all cell lines investigated (**Fig. 1**). No positive cell staining was observed when cells were incubated under identical conditions with a CF-labeled peptide consisting of the same amino acids, but in scrambled order (data not shown).

### Specificity of TPP binding to Hsp70

**Figure 2:**
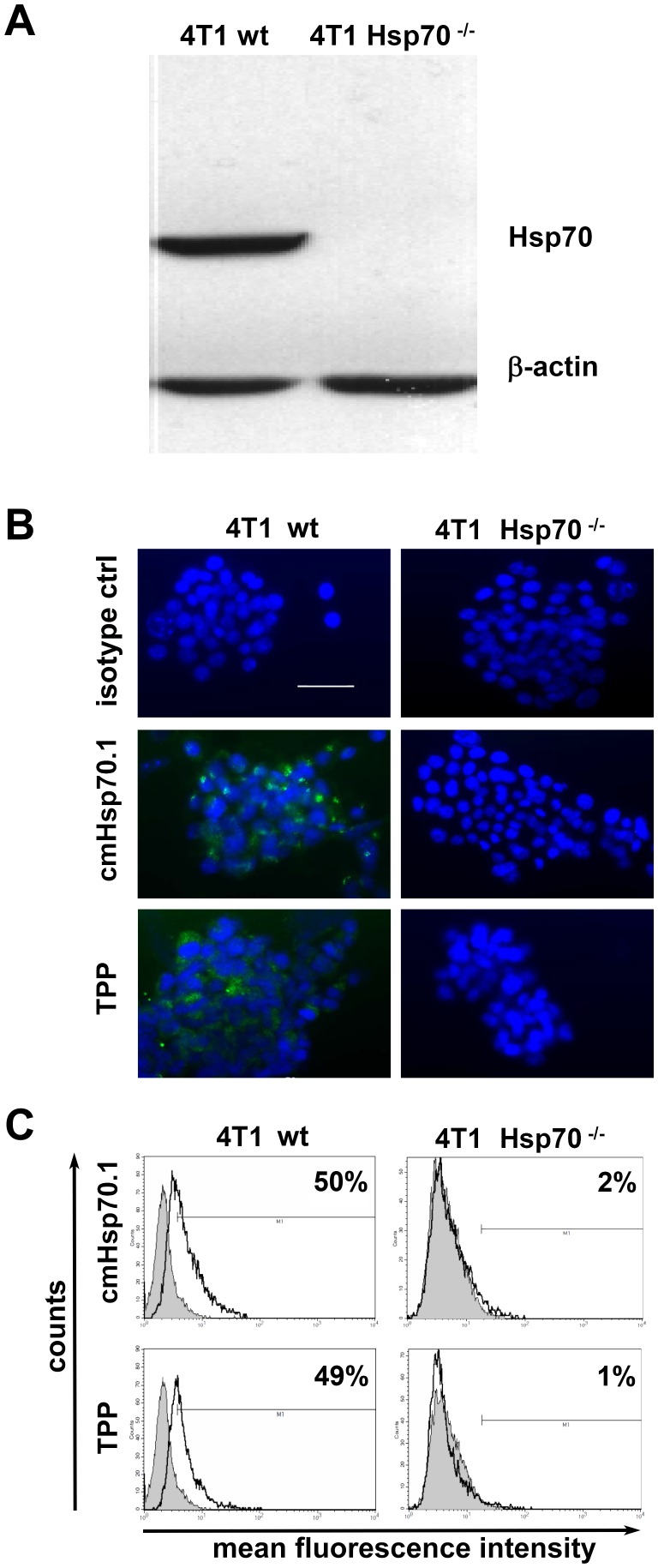
TPP specifically binds to Hsp70 *in vitro*. **A.** Western Blot analysis of whole cell lysates of 4T1 Hsp70 wild type (4T1 wt) and 4T1 knockout (4T1 Hsp70^−/−^) cell lines showed a positive Hsp70 staining in 4T1 wt but not in 4T1 Hsp70^−/−^ cells using the cmHsp70.1 antibody; ß-actin was used as a loading control. **B.** Immunofluorescence staining and **C.** flow cytometric analysis of *in vitro* grown viable 4T1 wt and Hsp70^−/−^ cells proved the Hsp70 specificity of CF-labeled TPP. Binding of TPP to 4T1 wt cells was comparable to that of cmHsp70.1 antibody (left panel, green staining). Neither TPP nor cmHsp70.1 antibody did bind to 4T1 Hsp70^−/−^ cells (right panel). Cells were counter-stained with DAPI (blue staining). **C.** Representative flow cytometric histograms indicate a positive Hsp70 staining of 4T1 wt cells by cmHsp70.1 antibody and TPP. In contrast, 4T1 Hsp70^−/−^ cells were neither stained with cmHsp70.1 antibody nor with TPP; grey, isotype controls, open histograms, Hsp70 specific reagents. The numbers in the histograms show the proportion of Hsp70 membrane-positively stained cells.

Knockout experiments revealed that Hsp70 was present in whole cell lysates of the 4T1 wild type (4T1 wt) cell line, but not in the Hsp70 knockout cell line 4T1 Hsp70^−/−^, as determined with the Hsp70 specific monoclonal antibody cmHsp70.1 (**Fig. 2A**) and SPA810 antibody (data not shown). Specific binding of CF-labeled TPP to memHsp70 was proven by immunofluorescence staining (**Fig. 2B**) and flow cytometric analysis (**Fig. 2C**). CmHsp70.1-FITC antibody as well as TPP showed specific staining of viable 4T1 wt cells (**Fig. 2B**, left panel), but not of 4T1 Hsp70^−/−^ cells (**Fig. 2B**, right panel). These data were confirmed by flow cytometric analysis. The proportion of Hsp70 positively stained 4T1 wt cells was comparable after incubation with cmHsp70.1-FITC antibody and TPP (**Fig. 2C**, upper panel). In contrast, 4T1 Hsp70^−/−^ cells did not show any staining with both Hsp70 reagents (**Fig. 2C**, lower panel).

**Figure 3:**
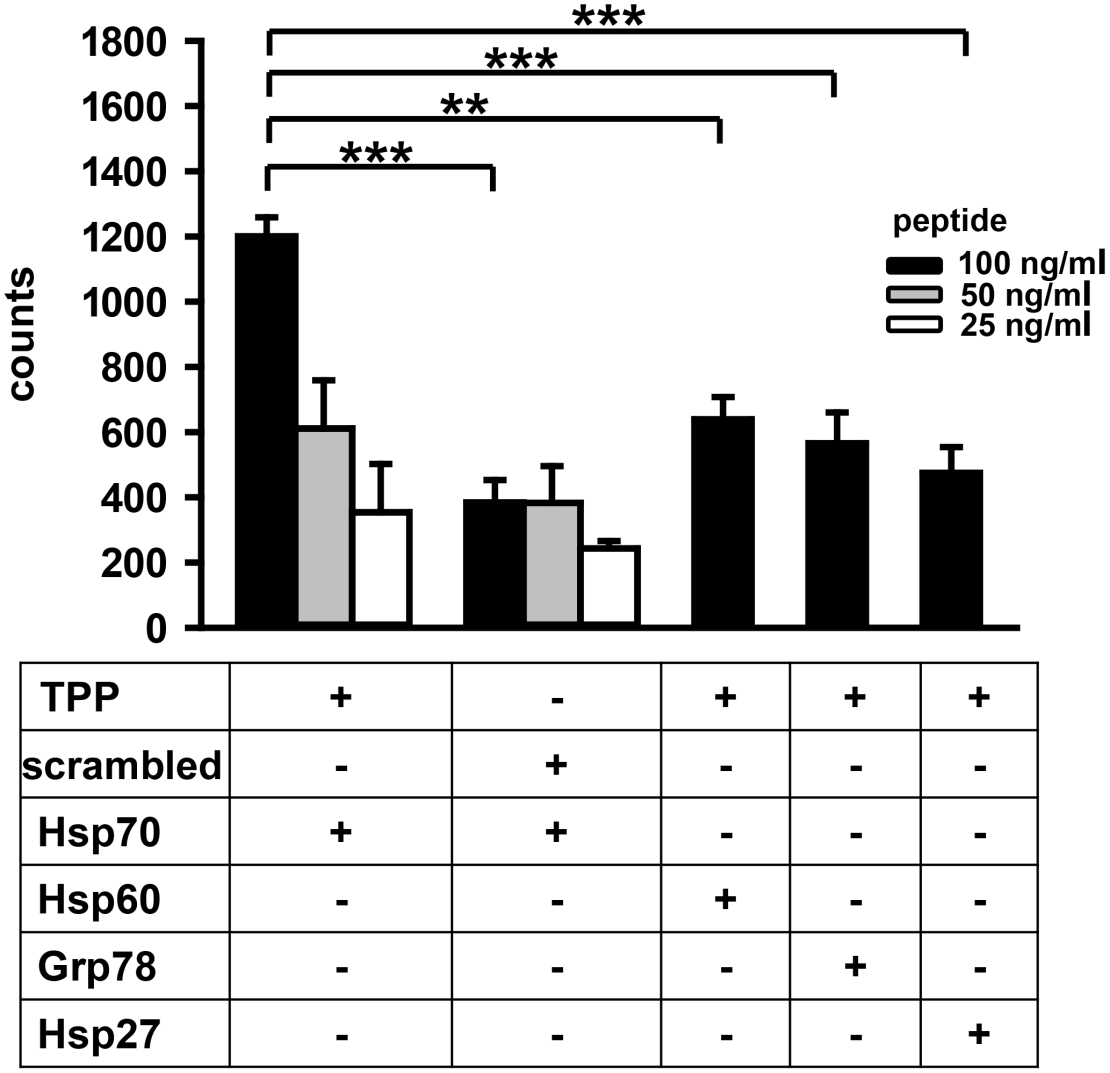
TPP specifically binds to Hsp70, but not to other Heat shock proteins (HSPs). A peptide ELISA was performed to determine the *in vitro* binding capacity of peptides to different HSPs. Ninety six well plates that were coated either with Hsp70, Hsp60, Grp78, or Hsp27 were incubated with carboxyfluorescein (CF)-labeled TPP or a scrambled peptide at concentrations ranging from 100 to 25 ng/ml. Fluorescence was measured after 30 min at 27°C using a multiplate reader. Combinations of peptides and proteins are indicated as “+” in the lower panel. Differences in peptide binding (100 ng/ml) were evaluated using the Student’s *t*-test (** *p*<0.01; *** *p*<0.001) (n = 3).

The binding capacity of TPP to different heat shock proteins was assessed using a peptide enzyme linked immunoassay (ELISA). For this, Hsp70, Hsp60, Grp78, or Hsp27-coated plates were incubated with CF-labeled TPP or scrambled peptide (27°C for 30 min) at the indicated concentrations (100, 50, 25 ng/ml) and the resultant fluorescence was measured using a multilabel reader. A strong concentration- and time-dependent signal was detectable when TPP was incubated with Hsp70-coated microtiter plates (**Fig. 3** left hand bars; 1,200 ± 154 counts). Peptide binding to Hsp70 was specific, as only a very weak background binding of scrambled peptide was observed (384±119 counts). Furthermore, although there was some interaction between the highest concentration of TPP (100 ng/ml) and Hsp60-, Grp78- or Hsp27-coated microtiter plates (638±154, 566±189, 474±177 counts, respectively, **Fig. 3**, right hand bars), other concentrations of TPP resulted in signals that were similar to those measured following incubation with the scrambled peptide (384±119 counts) (**Fig. 3**, left hand bars).

### Internalization of TPP and its relationship with the memHsp70 status

**Figure 4:**
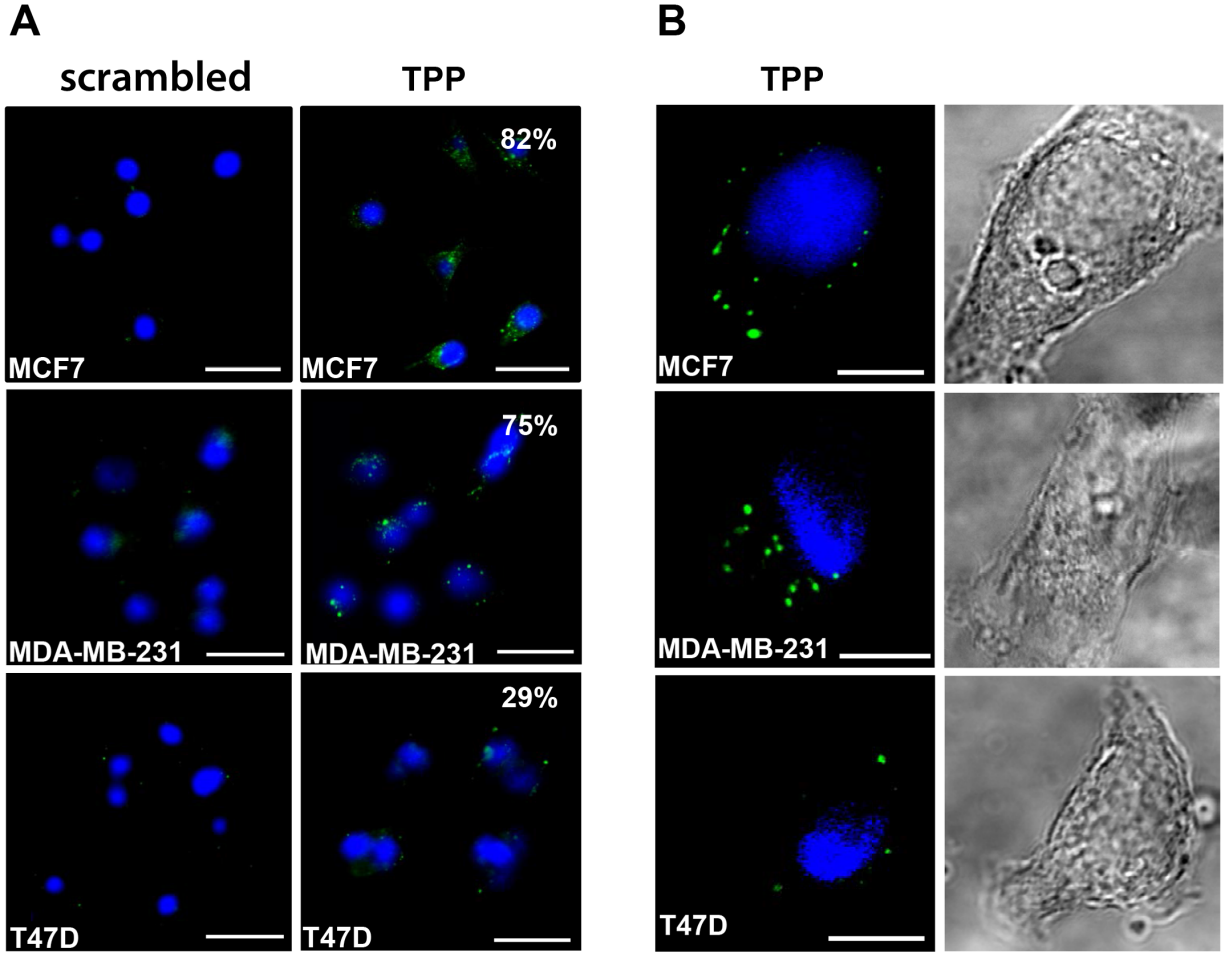
Specific uptake of TPP into tumor cells at 37°C. Human breast cancer cell lines expressing different levels of memHsp70 (MCF7, MDA-MB-231, T47D) were incubated with CF-labeled TPP or a scrambled peptide for 30 min at 37°C and the internalization of peptides imaged using confocal microscopy. **A.** TPP (green, right panel), but not the scrambled peptide (left panel) is internalized into the tumor cell lines. The appearance of the TPP (green, right panel) in well-defined, localized points suggests that the association of the peptide with intracellular vesicles is related to the memHsp70 expression status. The numbers shown as inserts indicate the percentage of memHsp70 positively stained cells. DAPI staining allows visualization of the nucleus (blue). Objective 2×; scale bar 50 µm. **B.** Individual cells were imaged as a z-stack in order to better illustrate the intracellular localization of TPP. Images are representative three-frame composites from approximately mid-way in the stack. Immunofluorescence staining, (left panel); brightfield, (right panel). Objective 63×; scale bar 10 µm.

Since the specific binding of TPP to Hsp70 could be shown using the ELISA (**Fig. 3**) and the flow cytometry analysis of tumor cell lines (**Fig. 1**), the dependency of peptide binding is dependent on the Hsp70 membrane expression of cells, the internalization of peptide into tumor cells was assessed using a panel of breast cancer cell lines which exhibit differences in their Hsp70 membrane expression profiles. A lower proportion of T47D cells exhibit memHsp70 expression in culture (∼29%), as compared with MDA-MB-231 and MCF7 cells (75% or 82%, respectively) (**Fig. 1**). The internalization of TPP by MCF7, MDA-MB-231 and T47D cells was examined by confocal microscopy using a Zeiss 510 inverted confocal microscope (**Fig. 4**). Initially, tile scans using an open pinhole on the microscope to maximize detected light were produced in order to provide an overview of the internalization (**Fig. 4A**). Incubation with TPP resulted in green fluorescent spots in all of the human breast tumor cell lines (**Fig 4A**, right column), whereas no internalization of the scrambled peptide could be detected (**Fig 4A**, left column). The appearance of the peptide (green) in well-defined areas suggests that it is not randomly distributed within the cytoplasm, but that it is associated with intracellular vesicles. The representative images shown were taken through a 20× objective and subjected to brightness, contrast and noise processing in ImageJ. The internalization of CF-labeled TPP was confirmed by creating z-stacks of individual cells (**Fig. 4B**). MCF7 and MDA-MB-231 cells, which express high amounts of Hsp70 on their plasma membrane, contained a large amount of green fluorescent spots (**Fig. 4B**, left column, top and middle panel), whereas the internalization of TPP by T47D cells was much less pronounced (**Fig. 4B**, left column, bottom panel). Images shown are representative three-frame composites from approximately mid-way up the stack. Brightfield images (**Fig. 4B**, right column) confirmed that imaged cells were morphologically healthy/normal.

### Rapid uptake of TPP into memHsp70+ tumor cells

**Figure 5:**
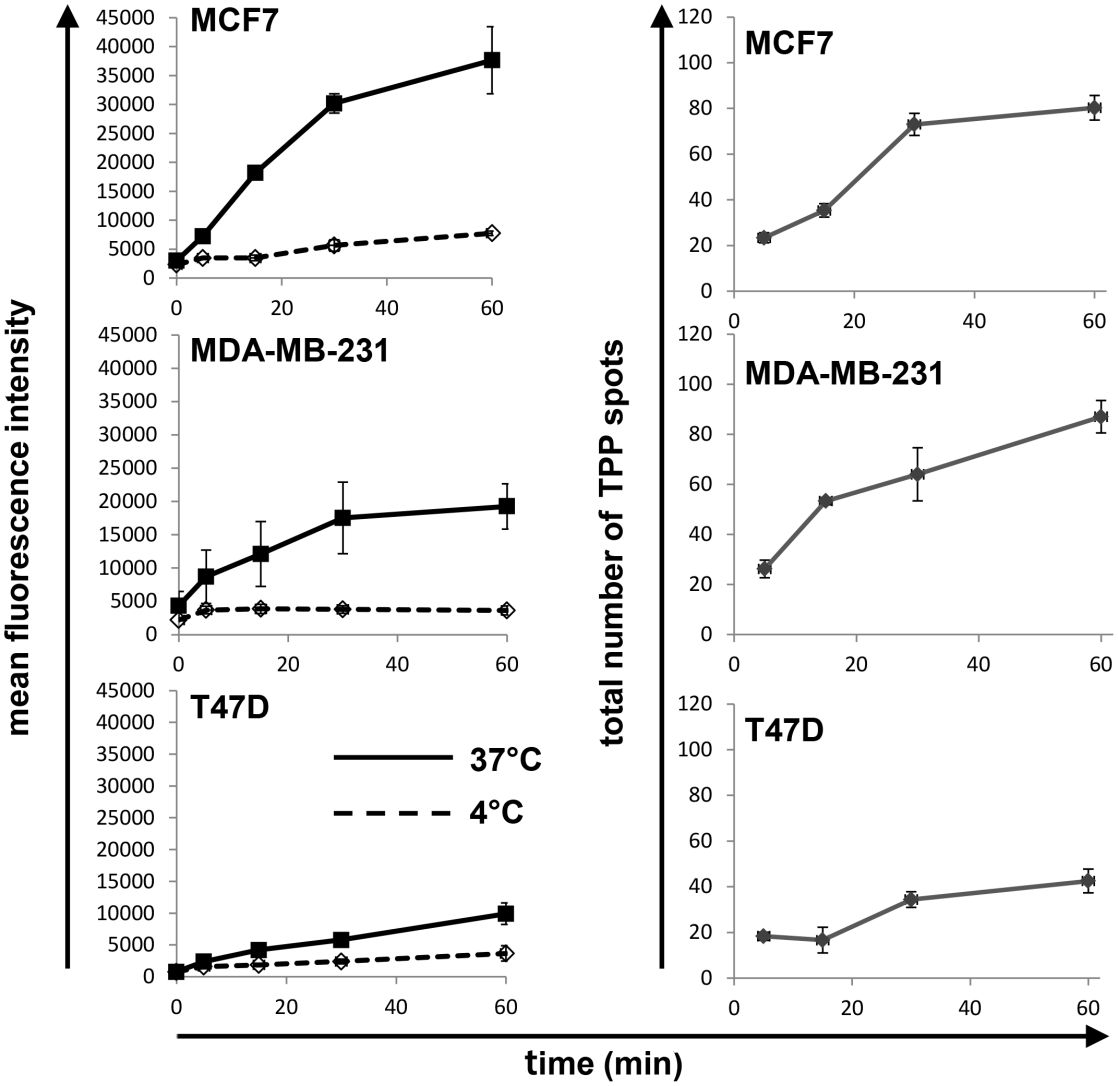
Kinetics of TPP internalization. (Left panel) Flow cytometric analysis of memHsp70 positive human breast cancer lines MCF7, MDA-MB-231, T47D reveals an accumulation of the fluorescence intensity after incubation with CF-labeled TPP at 37°C between 1 and 60 min (solid lines). At 4°C (dashed lines) mean fluorescence intensity remained low within the same time frame. Cell lines with a high percentage of Hsp70 membrane positive cells (MCF7, MDA-MB-231) exhibit a higher and more rapid uptake of TPP, whereas the cell line with low Hsp70 membrane expression (T47D) exhibits only a low uptake of TPP. (Right panel) Confocal microscopy images were analyzed to provide a total count of fluorescent spots per cell after incubation with the TPP at 37°C for 30 min. Although fluorescent spots progressively accumulated in all three cell lines, this was most apparent in the MCF7 and MDA-MB-231 cell lines that express higher levels of memHsp70 than T47D cells.

To further quantify the uptake of CF-labeled TPP, the internalization was monitored using flow cytometry. For these experiments, cells were incubated with the peptide for up to 60 min at either 37°C (to allow internalization, solid lines) or 4°C (to allow surface binding only, dashed lines) (**Fig. 5**, left column). The uptake of TPP is seen as an increase of the mean fluorescence intensity (mfi) of the cell population over time. At 4°C there is only surface binding and no progressive increase in the fluorescent intensity, with the mfi of all cell lines being less that 5,000 (**Fig. 5**, left column, dashed line). The cell lines with the highest expression of memHsp70 exhibited the greatest uptake of TPP (MCF7: mfi of 37,637±5,800 after 60 min and MDA-MB-231: mfi of 19,261±3,408 after 60 min; **Fig. 5** left column, top and middle panels). The cell line with the lowest expression of memHsp70 (T47D) exhibited the lowest uptake of TPP (mfi 9,900±1,358 after 60 min; **Fig. 5**, left column, bottom panel). The half-maximum time points are approximately 20 min for all three cell lines (data not shown).

The number of fluorescent spots per cell was also manually counted. The cell lines expressing the highest level of memHsp70 (MCF7, MDA-MB-231) contained around 80 fluorescent TPP spots per cell after 60 min (**Fig. 5**, right column, top panels), whereas the number of spots in the T47D cells was around 50% of this (**Fig. 5**, right column, bottom panel). Again half-maximum time-points for the internalization signal were around 20 min for all three cell lines (data not shown).

Taken together, these data demonstrate a rapid memHsp70 dependent uptake of TPP into breast cancer tumor cells.

### Internalization of TPP by memHsp70 expressing cells preferentially occurs at the cell adhesion interface

**Figure 6:**
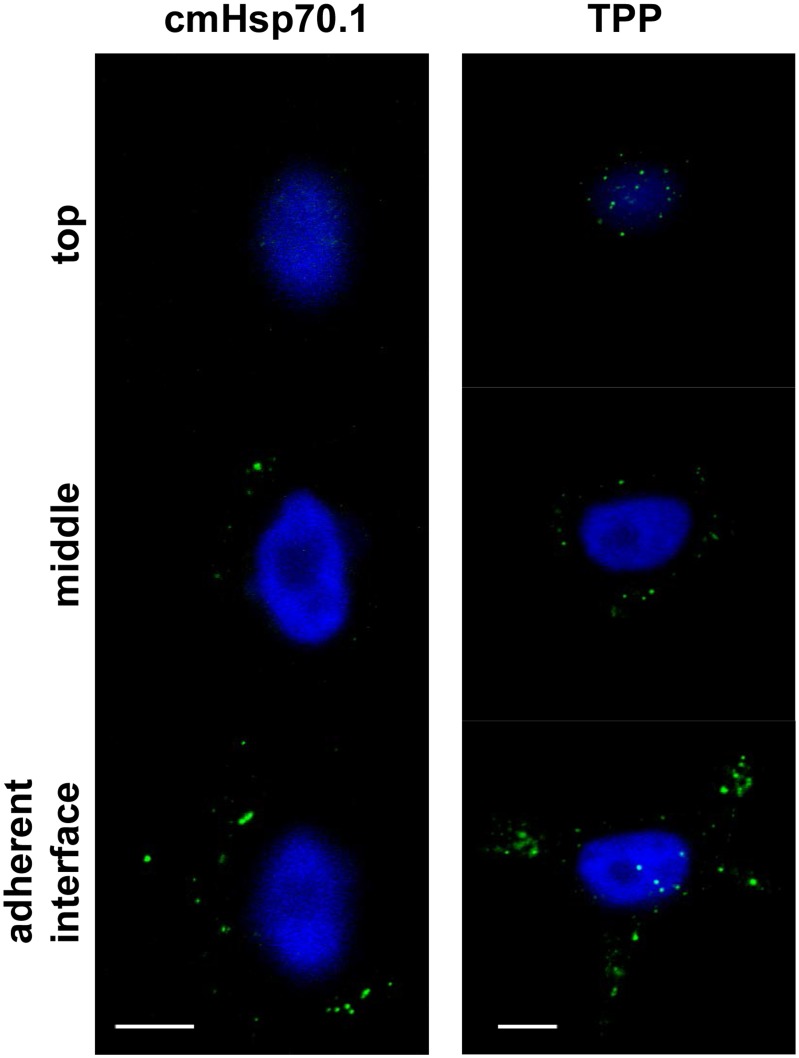
Internalization of TPP and cmHsp70.1 antibody via memHsp70 preferentially occurs at cellular adhesion points. Fluorescent vesicles are more prevalent towards the adherent interface of MCF7 cells after 30 min incubation at 37°C with cmHsp70.1 antibody (left panel) or TPP (right panel). Images are representative three-frame composites from the adherent surface, mid-way up the cell and at the apex. Objective 63×; scale bar 10 µm.

Across all tumor cell lines and all time points, the greatest number of fluorescent spots was present in the first quartile of the distance between the adherent and non-adherent surfaces of the cell. However, brightfield imaging of the cells (**Fig. 4B**, right column) did not allow accurate measurements of the cell size to be made, and the capacity to definitively interpret the data by normalizing spot number to cross-sectional area is therefore limited. Confocal fluorescence imaging shows that the green spots were visible in MCF7 cells after 30 min incubation with CF-labeled TPP (**Fig. 6**). The number of green spots increases as the focusing plane moves from the top to the bottom (adherent surface) of the cells (**Fig. 6**, from the top down to the bottom panels). Furthermore, there is no obvious difference in staining of FITC-labeled cmHsp70.1 (**Fig. 6**, left column) compared to TPP (**Fig. 6**, right column). Images shown are representative three-frame compositions, from the point of adherence, halfway up the cell and the apex (objective 63, scale bar 10 µm). Similar staining patterns were also observed for MDA-MB-231 and T47D cells (data not shown).

**Figure 7:**
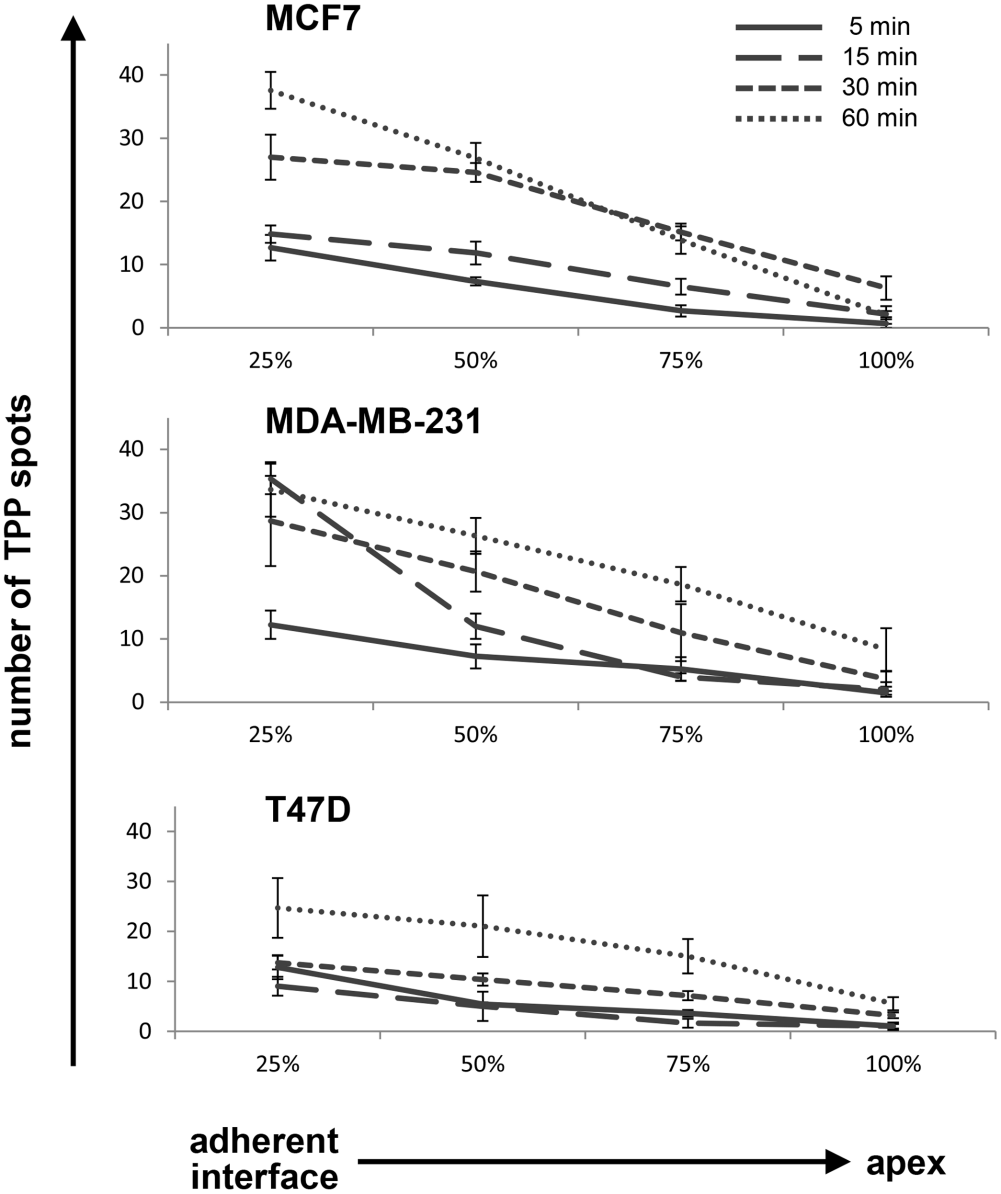
Localization of internalized TPP within cells. Confocal microscopy z-stacks were used to divide cells into quartiles using percentages of cell height from the adherent interface (0%) to the apex of the cells (100%). The number of fluorescent TPP spots in each quartile was counted in order to quantify the peptide distribution. For all time points and all cell lines investigated, TPP spots were found to be more prevalent in the quartile which included the adherent surface. This finding suggests that TPP is preferentially internalized and trafficked via this interface. The data shown are representative composite images of every z-stack frame counted in each quartile. Images were produced using a 63×/1.4 oil immersion lens on an inverted Zeiss 510 confocal microscope.

We further evaluated the preferential, intracellular location of internalized CF-labeled TPP by developing an approach to quantify the number of TPP spots throughout the cell. Using confocal microscopy derived z-stacks cells were divided into quartiles from the adherent surface (0%) of the cell to its apex (100%). The number of fluorescent spots in each quartile was counted in order to quantify the localization of TPP within the cells. The maximum number of fluorescent spots (vesicles) at the adherent surface after 60 min was 38±3 in MCF7 cells (**Fig. 7**, top diagram), 34±4 in MDA-MB-231 cells (**Fig. 7**, middle diagram), and 24±6 in T47D cells (**Fig. 7**, bottom diagram). For all time points and all cell lines investigated, the greatest number of fluorescent spots was present in the quartiles adjacent to the adherent surface (MCF7 *p*<0.001, MDA-MB231 *p*<0.01, T47D *p*<0.05, Factorial ANOVA). These findings indicate that TPP is continually internalized via this interface and confirm that there a time-dependent uptake and accumulation of TPP in distinct intracellular vesicles which appears to preferentially occur at the adherent interface.

### Endosomal vesicles show distinct patterns in MCF7 cells

**Figure 8:**
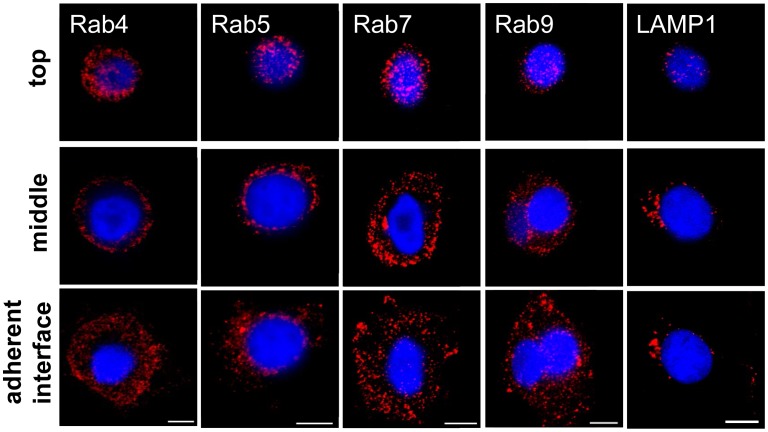
Distribution of intracellular vesicles in MCF7 cells. MCF7 tumor cells grown in glass-bottomed MatTek dishes for 48 h were fixed in paraformaldehyde, permeabilized and stained with primary antibodies specific for early (Rab4, Rab5), late (Rab7, Rab9) and recycling (Rab11) endosomes or lysosomes (LAMP1), followed by an appropriate Cy3-labeled secondary antibody (red). The nucleus was counter-stained with DAPI (blue). Representative three-frame composites from across the z-stack are shown. All of the vesicular markers assessed were found to be homogenously distributed throughout the cells. Objective 63×; scale bar 10 µm.

As the distinct fluorescent patterning of internalized CF-labeled TPP is suggestive of its association with intracellular vesicles, one possible explanation for the greater accumulation of TPP close to the adherent surface of the cells would be that intracellular vesicles are also preferentially localized to this region of the cell. In order to examine this potential explanation, MCF7 cells were grown in glass-bottomed MatTek dishes for 48 h, at which time they were fixed in paraformaldehyde, permeabilized and stained with primary antibodies that were specific for early endosomes (Rab4, Rab5), late (Rab7, Rab9) endosomes or lysosomes (LAMP1), followed by an appropriate Cy3-labeled secondary antibody (**Fig. 8**). The vesicular marker proteins showed a widespread distribution throughout the cell, and it was not evident that any given vesicle population was highly localized, particularly near the adherent surface (**Fig. 8** **)**.

### Uptake of TPP via memHsp70 follows an endosomal pathway

**Figure 9:**
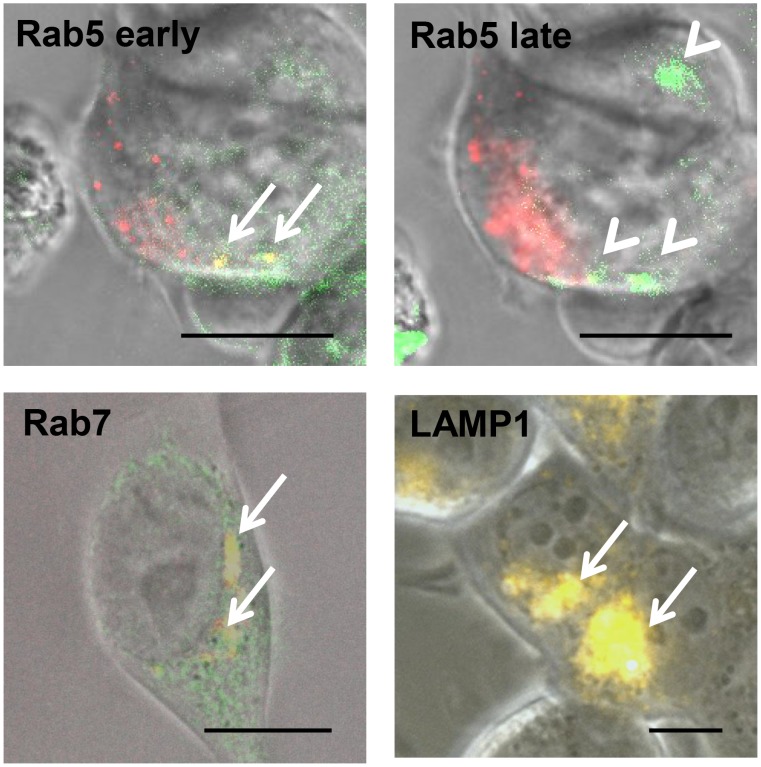
Uptake of TPP between 0 and 60 min follows an endosomal pathway. MCF7 cells take up CF-labeled TPP via an endosomal transport route in a time-dependent manner. Co-localization of TPP (green) with the endosomal marker proteins (Rab5, Rab7, LAMP1; each in red) is visible as a yellow signal. Co-localization of TPP with Rab5 can be seen within early endosomes at early time points (<30 min, arrows, upper left graph), but not at later time points (arrow heads, upper right graph). Between 30 and 60 min, TPP co-localizes with Rab7 vesicles (arrows, lower left graph). After 60 min, TPP co-localizes with LAMP1 positive lysosomes (arrows, lower right graph). Images are representative three-channel composites: brightfield, FITC, RFP. Objective 63×; scale bar 10 µm.

The apparent uniform distribution of endosomal and lysosomal vesicles throughout the cells provides no insight into the final trafficking of CF-labeled TPP. Difficulties were encountered when trying to co-stain cells that had been incubated with TPP with antibodies to endosomal and lysosomal markers (possibly as a result of peptide leakage from permeabilized cells). Therefore, cells were transfected with vectors expressing red fluorescent protein (RFP)-labeled marker proteins in order to identify different vesicles - Rab5 for early endosomes, Rab7 for late endosomes and LAMP1 for lysosomes. Rab5 staining reveals small vesicles near the plasma membrane (**Fig. 9**, upper left graph). Vesicles expressing Rab7 localize between plasma membrane and nucleus (**Fig. 9** lower left graph). LAMP1 staining is detectable in large structures with perinuclear localization (**Fig. 9** lower right graph).

MCF7 cells took up TPP via endosomal transport routes in a time-dependent manner. Co-localization of TPP (green) with early and late endosomal and lysosomal marker proteins (Rab5, Rab7, LAMP1; each in RFP) was visible as a yellow signal. Co-localization of TPP with Rab5 (early endosomes) was detected at time points <30 min (**Fig. 9**, upper left graph). At later time points (<30 min), the yellow signals disappeared, leaving green staining for TPP and red staining for Rab5 (**Fig. 9**, upper right graph). Subsequently (between 30 and 60 min), co-staining of TPP occurs with Rab7 vesicles (**Fig. 9** **,** lower left graph). TPP is co-localized with LAMP1 vesicles at time points >60 min, indicating transport of TPP into lysosomes (**Fig. 9**, lower right graph).

### Internalized TPP co-localizes to the mitochondria after 90 min

**Figure 10:**
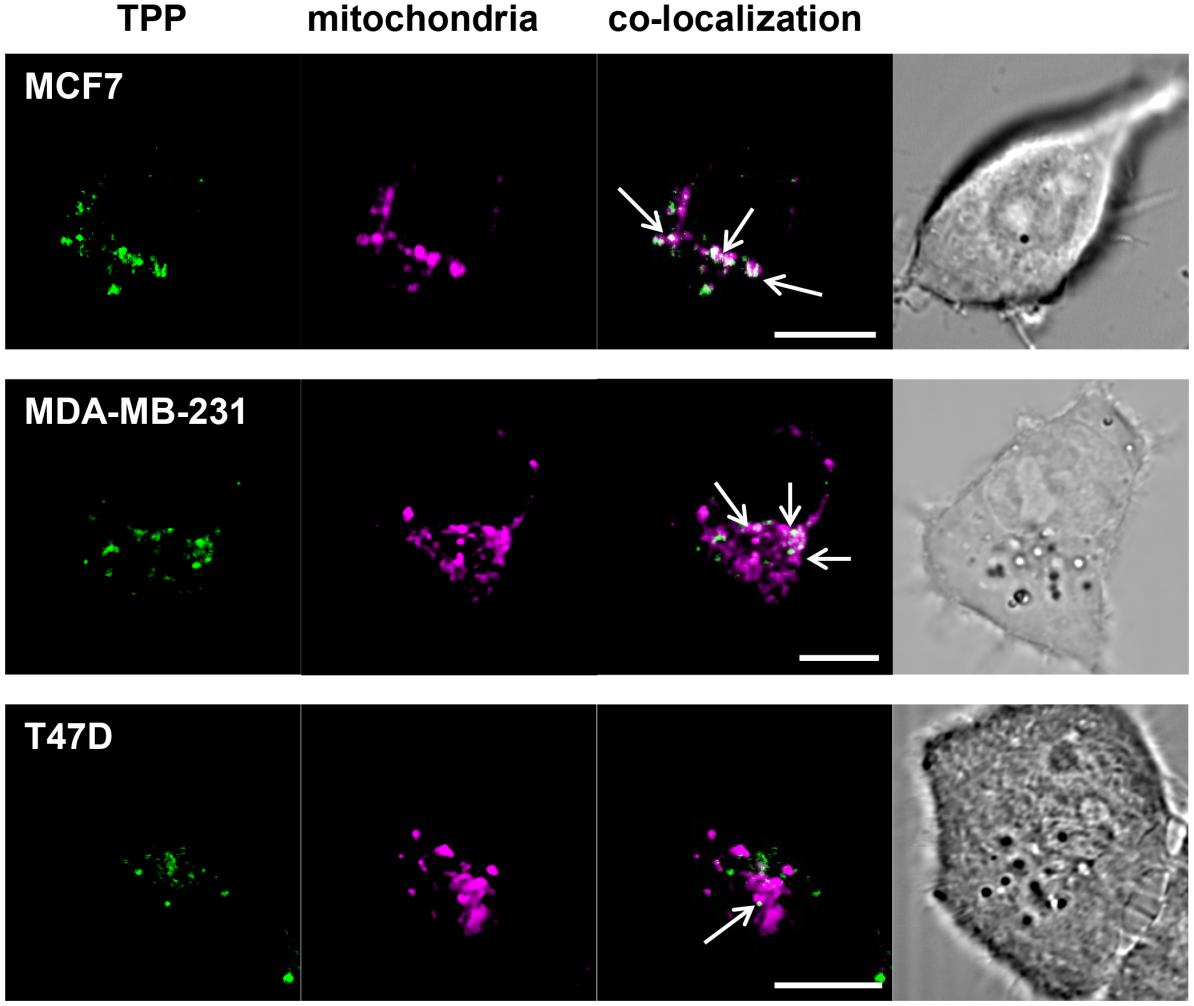
TPP co-localizes with mitochondria after 90 min. MCF7, MDA-MB-231, and T47D cells were incubated with CF-labeled TPP (green) for 90 min, stained with the mitochondrial detection dye Mito-ID (purple) and then imaged using confocal microscopy. A proportion of internalized TPP co-localizes with mitochondria in all three tumor cell lines (Pearson’s coefficient: MCF7 r = 0.855, MDA-MB-231 r = 0.585, T47D r = 0.813). The Manders’ M1 coefficient was used to estimate the proportion of total TPP that co-localizes with mitochondria (40% in MCF7 cells, M1 = 0.407; 44% in MDA-MB-231 cells, M1 = 0.443). In contrast, 14% of TPP was co-localized to mitochondria in T47D cells (M1 = 0.141). These findings correlate with the differential Hsp70 membrane expression levels of the respective cancer cell lines. Images are representative single frames. Objective 63×; scale bar 10 µm.

In order to measure whether internalized TPP (green) localizes to mitochondria, MCF7, MDA-MB-231 and T47D cells were incubated with TPP and co-stained using the mitochondrial detection dye Mito-ID (magenta) (**Fig. 10**). Cells were imaged using confocal microscopy and subsequent co-localization statistics confirm that TPP localizes to the mitochondria in all three cell lines. The Pearson’s r coefficients for MCF7, MDA-MB-231 and T47D cells were 0.855 (**Fig. 10**, top row), 0.585 (**Fig. 10**, middle row) and 0.813 (**Fig. 10**, bottom row) respectively. An estimation of the proportion of the total TPP that was co-localized with the mitochondria was obtained by calculating the Manders’ M1 coefficient. Using this approach, 40–45% of TPP localized to the mitochondria in MCF7 and MDA-MB-231 cells (MCF7, M1 = 0.407, MDA-MB-231, M1 = 0.443), whereas only 14.1% of the TPP co-localized with mitochondria in T47D cells (M1 = 0.141). Again, this correlates with the memHsp70 expression profile of the cells.

## Discussion

Breast cancer is the most common tumorigenic malignancy for women in Western countries and is the primary cause of mortality [38], [39]. Immunotherapy and targeting mammary-specific targets using antibodies or small molecules coupled with cytotoxic substances offer a good adjunct to standard protocols including surgery, cytotoxic drugs, endocrine therapy and radiation therapy [40]. Although new drugs are in development [41], the heterogeneity of cancer resulting from mutated genes, the differential expression of specific surface molecules, and/or the status of patients with regards to the stage and subtype of disease, makes it difficult to develop molecules and agents with broad specificity across individual patient groups [42]. Interest in the use of peptides as agents for imaging and the specific delivery of therapeutic agents to tumors is therefore growing [11], [43]–[46].

Peptides offer a number of advantages such as better biodistribution profiles and a greater ability to penetrate tissues. The efficacy of peptides for anti-cancer therapies is often dictated by their ease of binding and uptake into tumor cells. As examples, cathelicidin is been a potential therapeutic peptide for gastrointestinal inflammation and cancer [47] and a 15-mer peptide from the follicle-stimulating hormone support the anti-tumor activity of paclitaxel nanoparticles against ovarian cancers [48]. The cancer-specific peptide BR2 penetrates cancer cells, and has been shown to mediate the delivery of a scFv into cancer cells [49]. The therapeutic capabilities of peptides have been demonstrated by a report of a peptide which is able to induce apoptosis in SKOV3 cells by down-regulating Bcl-2 [50]. The kinetics of peptide uptake has been studied in lymphocytes and monocytes [51].

Interestingly, peptides have also been shown to facilitate the delivery of larger molecules. Nanostructured lipid carriers bound to small 5-mer peptides are taken up by EGFR-overexpressing tumors *in vivo* [52] and such peptides might therefore support the targeted delivery of chemotherapeutic agents. Novel peptides are also being used as targeted drug delivery systems for breast cancer therapy and also for tumor imaging strategies [53]. A telomerase-derived peptide has been shown to increase the cytosolic delivery of macromolecules by heat shock mediated cell penetration [54]. Other studies support the use of peptides as lead compounds for anti-cancer therapy. For example a yeast two-hybrid screening has identified peptide aptamers which bind to Hsp70 and specifically inhibit chaperone activity, thereby increasing sensitivity to drug-induced apoptosis [55].

For the first time we have shown that a 14-mer peptide TPP derived from the native Hsp70 protein can specifically recognize and target tumor cells expressing the membrane form of Hsp70. Given the selective, but widespread, expression of memHsp70 on tumor cells, but not their non-malignant counterparts [14], [56]–[58] appropriately formulated TPP could offer a promising new clinically relevant small molecule for imaging and/or specifically targeting tumors. TPP has advantages over other peptides that are currently being evaluated, as the latter are restricted to targeting specific receptors on specific types of tumor cells [7]–[11]. Another potential advantage of TPP as a therapeutic vehicle is that memHsp70 is expressed on a large proportion of tumors and its expression on tumor cells can be induced/increased using relevant chemotherapeutic agents [59] and radiation therapy [60]. The proportion of patients to which the TPP can be administered can therefore be increased by standard therapies.

Fluorescence microscopy has previously revealed the internalization of Hsp70 and granzyme B (GrB) into the CT26 murine colon cancer cell line involves Rab and LAMP dependent vesicles [30], and we therefore anticipated that the internalization of TPP also involves an endosomal pathway which is associated with Rab proteins inside tumor cells. Endosomes are intracellular vesicles that are responsible for the transport of molecules between different intracellular compartments [61], and they can be described as early endosomes (EE), late endosomes (LE), and recycling endosomes (RE) [62]. The endosomal vesicles are also linked to the endoplasmic reticulum and the trans-Golgi network [62]. Lysosomes are thought to collaborate with endosomal transport routes to generate final vesicles in which molecules are deconstructed or catabolized [63], [64]. Monomeric GTPase proteins (Rab) [61] within the membranes of endosomes are responsible for transport, docking, and merging, and these can also serve as targets for imaging. Currently, around 40 different Rab proteins have been described in mammals. As typical marker proteins, Rab5 is described for early endosomes [65], Rab7 for late endosomes [65], [66], and Rab11 for recycling endosomes [67], [68]. Lysosome associated proteins (LAMP) are integral membrane proteins which stabilize lysosomal membranes [69], with both LAMP1 and LAMP2 being typically used for identifying lysosomes [64].

Gold-nanoparticles coated with pro-apoptotic peptides can damage mitochondria [70] and gold-peptide nano-assemblies targeting mitochondria have been shown to have more pronounced cancer cell killing properties [71]. Alternatively, ionidamine liposomes can be used to enhance the treatment of drug-resistant cancers by acting on mitochondrial signaling pathways [72], or by delivering the redox cycler doxorubicin, as a source of ROS production, to cancer cell mitochondria [73]. Retinoblastoma protein directly induces apoptosis at the mitochondria [74]. Cytotoxic drugs can exert their apoptotic function at the level of the mitochondria, as has been illustrated by the direct influence of Doxorubicin on mitochondria-initiated apoptosis [75]. The mechanisms of action have been elucidated using a number of different approaches, an example of which has been observing the translocation of a peptide through mitochondrial membranes using NMR observing the translocation of a peptide through mitochondrial membranes [76]. Other peptides show anti-tumor activity by directly targeting the mitochondrial membrane and inducing apoptosis [77], [78]. With regards to TPP, to date we have been unable to detect a direct apoptotic activity on the basis of cytochrome c release or active caspase-3 staining in breast cancer cell lines at concentrations up to 750 µg/ml and up to 72 h after incubation (data not shown). Targeting the mitochondrial membrane might be useful for the optical imaging of tumors, including breast cancer cells in xenograft mouse models [79]. We are therefore investigating the use of TPP as an imaging and therapeutic agent in murine tumor models (manuscript in preparation).

In summary, this study demonstrates the specific binding and internalization of TPP by different cancer cells expressing the membrane form of Hsp70. TPP might therefore have potential as an agent for targeting and imaging the large proportion of tumors (∼50%) which express Hsp70 on their plasma membrane.
